# Supplementary material for: From the perspective of rumen microbiome and host metabolome, revealing the effects of feeding strategies on Jersey Cows on the Tibetan Plateau
Source: PeerJ. 2023 Sep 11;11:e16010. doi: 10.7717/peerj.16010 (PMC10501371; doi:10.7717/peerj.16010)
Supplement: Data S1 [file peerj-11-16010-s001.doc]

**Table S1** Composition and energy of the TMR, grazed pasture and concentrate (on dry matter basis) %.

| Items | TMR | Pasture | Concentrate |
| --- | --- | --- | --- |
| Corn silage | 35.25 |  |  |
| Highland barley straw | 14.38 |  |  |
| Oat Hay | 15.37 |  |  |
| Corn | 18.15 |  | 51.86 |
| Soybean meal | 10.95 |  | 31.29 |
| Wheat bran | 4.50 |  | 12.86 |
| Stone powder | 0.45 |  | 1.29 |
| NaCl | 0.35 |  | 1.00 |
| CaHPO_4_ | 0.10 |  | 0.29 |
| Premix^1)^ | 0.50 |  | 1.43 |
| Total | 100 |  | 100 |
| Nutrient levels |  |  |  |
| NE（MJ/kg） | 6.04 | 4.52 | 7.90 |
| Crude protein (CP) | 12.17 | 7.00 | 19.82 |
| Neutral detergent fiber (NDF) | 43.66 | 44.24 | 14.70 |
| Acid detergent fiber (ADF) | 22.47 | 70.36 | 6.14 |
| Ca | 0.46 | 0.61 | 0.86 |
| P | 0.34 | 0.18 | 0.59 |

^1^The premix provided the following per kg of diets: VA 5 130 IU, VD 3 mg, β-carotene 0.10 mg, Mn 12 mg, P 12 mg, S 0.85 mg, Zn 64 mg, Se 0.4 mg, Co 0.19 mg. 1 283 IU, VE 26 mg.

**Table S2** Serum differential metabolites in positive and negative ionization modes

| **Adduct** | **Name** | **VIP** | **Fold change** | ***P* value** | **m/z** | **rt(s)** | **SuperClass** |
| --- | --- | --- | --- | --- | --- | --- | --- |
| [M+H]+ | Arg-Gly-Asp | 1.4025 | 10.5359 | 0.0000 | 347.1501 | 328.5690 | Organic acids and derivatives |
| [M+H]+ | Trans-4-(aminomethyl)cyclohexanecarboxylic acid | 8.7308 | 0.0339 | 0.0000 | 158.1177 | 339.9730 |  |
| [M+H]+ | L-homocitrulline | 6.7718 | 4.0591 | 0.0000 | 190.0976 | 336.3880 |  |
| [M+H]+ | N-acetyl-dl-valine | 1.7378 | 0.3061 | 0.0001 | 160.0969 | 362.0065 |  |
| [M+H]+ | Stachydrine | 7.5258 | 0.2131 | 0.0002 | 144.1020 | 380.5110 |  |
| [M+H-C3H6O4]+ | Chorismic acid | 1.8883 | 0.3528 | 0.0002 | 121.0287 | 299.4730 |  |
| [M+H-NH3]+ | Lys-Ala | 1.5121 | 0.4117 | 0.0007 | 201.1169 | 369.7060 |  |
| [M+H]+ | DL-isoleucine | 6.0934 | 0.6575 | 0.0012 | 132.1021 | 338.2320 |  |
| [M+H]+ | Glufosinate | 1.3447 | 0.5579 | 0.0018 | 182.0483 | 352.4580 |  |
| [M+H]+ | Asn-Gln | 1.5432 | 0.6243 | 0.0019 | 261.1016 | 390.3560 |  |
| [M+H]+ | DL-Glutamic acid | 1.9505 | 1.4504 | 0.0019 | 148.0605 | 378.8870 |  |
| [M+H]+ | Phenylacetyl-l-glutamine | 2.5444 | 3.4284 | 0.0022 | 265.1182 | 299.3190 |  |
| [M+H]+ | Acetohydroxamic acid | 2.4879 | 2.6017 | 0.0029 | 76.0400 | 282.8650 |  |
| [M+H-C3H4O3]+ | N-acetyl-l-phenylalanine | 4.4986 | 0.7562 | 0.0034 | 120.0811 | 326.3390 |  |
| [M+H]+ | Phenylalanine | 4.2888 | 0.7602 | 0.0037 | 166.0864 | 326.3390 |  |
| [M+H]+ | Ergothioneine | 2.3254 | 0.3332 | 0.0057 | 230.0958 | 367.4920 |  |
| [M+H]+ | Betaine | 16.4111 | 1.2708 | 0.0065 | 118.0866 | 345.8575 |  |
| [M+H-H2O]+ | L-homoserine | 1.3523 | 1.8752 | 0.0078 | 102.0554 | 448.4520 |  |
| [M+H]+ | DL-threonine | 1.6740 | 0.4985 | 0.0080 | 120.0658 | 377.5230 |  |
| [M+H]+ | Leu-Leu | 1.8307 | 0.0532 | 0.0105 | 245.1647 | 107.6750 |  |
| [M+2H]2+ | Asn-Arg-Lys | 1.4573 | 0.5274 | 0.0167 | 209.1536 | 61.9452 |  |
| [M+H-H2O]+ | O-succinyl-l-homoserine | 1.8388 | 0.3383 | 0.0216 | 202.0862 | 277.9050 |  |
| [M+H]+ | Gamma-l-glutamyl-l-glutamic acid | 2.6216 | 1.5238 | 0.0267 | 277.0894 | 364.5180 |  |
| [M+H]+ | Methionine | 1.3703 | 0.7236 | 0.0311 | 150.0583 | 343.4090 |  |
| [M+H]+ | N-epsilon-methyl-l-lysine | 1.2830 | 1.6233 | 0.0421 | 161.1366 | 397.5130 |  |
| [M+H-H2O]+ | L-saccharopine | 2.2743 | 0.0253 | 0.0489 | 259.1442 | 211.0440 |  |
| [M+H]+ | D-glutamine | 4.7090 | 1.1761 | 0.0494 | 147.0765 | 387.0430 |  |
| [M+H]+ | Beta-echinenone | 8.7414 | 13.3564 | 0.0000 | 551.4243 | 60.2778 | Lipids and lipid-like molecules |
| [M+H]+ | 3-hydroxy-4-keto-gamma-carotene | 4.6658 | 12.9086 | 0.0000 | 567.4189 | 59.2545 |  |
| [M+H]+ | Alloxanthin | 3.3318 | 25.3565 | 0.0000 | 565.4037 | 56.1956 |  |
| [M+H-H2O]+ | N-palmitoyl-d-sphingosine | 1.3232 | 1.9134 | 0.0000 | 520.5091 | 63.4645 |  |
| [M+H]+ | N-(octadecanoyl)sphing-4-enine-1-phosphocholine | 3.6218 | 1.3653 | 0.0001 | 731.6064 | 270.4505 |  |
| [M-H2O+H]+ | Taurolithocholate | 1.0018 | 2.2355 | 0.0002 | 466.2986 | 219.1580 |  |
| [M+H-H2O]+ | 6-ketoprostaglandin f1-alpha | 1.7321 | 5.7612 | 0.0010 | 353.2474 | 283.4270 |  |
| [M]+ | (+)-alpha-tocopherol | 7.3218 | 0.7065 | 0.0011 | 430.3792 | 55.6405 |  |
| [M+H]+ | 1-(1z-hexadecenyl)-sn-glycero-3-phosphocholine | 5.4403 | 0.6319 | 0.0015 | 480.3449 | 275.3180 |  |
| [M+NH4]+ | Cholic acid | 8.2168 | 2.6717 | 0.0027 | 426.3213 | 294.9450 |  |
| [M+H-3H2O]+ | Ursocholic acid | 8.4316 | 2.8177 | 0.0029 | 355.2631 | 295.4030 |  |
| [M+H]+ | N-nervonoyl-d-erythro-sphingosylphosphorylcholine | 1.7969 | 2.3304 | 0.0039 | 813.6847 | 283.2070 |  |
| [M-H2O+H]+ | 3-alpha,7-beta,12-alpha-trihydroxy-5-beta-cholan-24-oic acid | 2.0998 | 2.4745 | 0.0042 | 391.2841 | 294.9900 |  |
| [M+H-C11H18O]+ | Retinene | 3.2342 | 0.6648 | 0.0042 | 119.0858 | 63.5584 |  |
| [M+H-C7H13NO2]+ | Dodecanoic acid, 12-[[(cyclohexylamino)carbonyl]amino]- | 1.2630 | 0.4178 | 0.0053 | 198.1852 | 61.9068 |  |
| [M+H]+ | Neoline/bullatine b | 1.3891 | 1.6654 | 0.0064 | 438.2977 | 280.5140 |  |
| [M+H]+ | Isovaleryl-l-carnitine | 2.9628 | 1.8258 | 0.0068 | 246.1701 | 317.6670 |  |
| [M+H]+ | L-propionylcarnitine | 3.5437 | 1.5105 | 0.0075 | 218.1388 | 340.4440 |  |
| [M+H]+ | Palmitamide | 1.2234 | 0.4603 | 0.0087 | 256.2633 | 62.7774 |  |
| [M+H]+ | Succinic acid N,N-dimethylhydrazide | 1.3149 | 1.5032 | 0.0108 | 161.0710 | 285.0060 |  |
| [M+H-2H2O]+ | Norethindrone | 1.8330 | 0.0287 | 0.0137 | 263.1754 | 167.9970 |  |
| [M+H]+ | 1-o-hexadecyl-2-o-(4z,7z,10z,13z,16z,19z-docosahexaenoyl)-sn-glyceryl-3-phosphorylcholine | 2.6718 | 0.7166 | 0.0200 | 792.5906 | 249.1895 |  |
| [M+H]+ | 7-alpha-hydroxy-4-cholesten-3-one | 1.5314 | 0.5885 | 0.0206 | 401.3413 | 60.6319 |  |
| [M+H-H2O]+ | 1,8-octanediol | 2.2796 | 0.7789 | 0.0211 | 129.1025 | 337.3690 |  |
| [M+H]+ | 3-hydroxybutyrylcarnitine | 1.1608 | 0.5336 | 0.0218 | 248.1492 | 361.5310 |  |
| [M+H]+ | 1,2-dihexadecanoyl-sn-glycero-3-phosphocholine | 2.4665 | 1.4670 | 0.0243 | 734.5694 | 258.8300 |  |
| [M+H]+ | Isobutyryl-l-carnitine | 2.4580 | 1.4292 | 0.0285 | 232.1544 | 326.8220 |  |
| [M+H]+ | 1,2-dipentadecanoyl-sn-glycero-3-phosphocholine | 1.2515 | 1.4902 | 0.0326 | 706.5383 | 259.1900 |  |
| [M]+. | beta-cryptoxanthin | 3.4753 | 1.6449 | 0.0327 | 552.4325 | 55.9943 |  |
| [M+H]+ | 1-pentadecanoyl-sn-glycero-3-phosphocholine | 4.2353 | 1.2613 | 0.0429 | 482.3245 | 281.4880 |  |
| [M+H]+ | Stearoylcarnitine | 1.3450 | 0.8200 | 0.0439 | 428.3886 | 57.2225 |  |
| [M+H]+ | Erucamide | 6.3170 | 0.6536 | 0.0444 | 338.3416 | 61.0663 |  |
| [M+H]+ | 5-aminovaleric acid betaine | 4.2476 | 1.5940 | 0.0499 | 160.1333 | 397.3560 |  |
| [M+H-H2O]+ | Trans-zeatin | 1.0700 | 5.1675 | 0.0000 | 202.0975 | 323.6050 | Organoheterocyclic compounds |
| [M+H]+ | Phenanthridine | 1.3881 | 0.0957 | 0.0000 | 180.0995 | 340.0080 |  |
| [M+Na]+ | 7-chloro-3-methylquinoline-8-carboxylic acid | 2.8027 | 1.9997 | 0.0000 | 244.0275 | 36.2324 |  |
| [M+H]+ | 5-methoxytryptophan | 1.5593 | 3.7355 | 0.0000 | 235.1077 | 330.4300 |  |
| [M+H-NH3]+ | Serotonin | 6.2674 | 3.0137 | 0.0000 | 160.0758 | 330.4300 |  |
| [M+H]+ | Cinoxacin | 2.3331 | 2.2645 | 0.0001 | 263.0737 | 364.1190 |  |
| [M+H]+ | Lumichrome | 1.2927 | 1.4688 | 0.0007 | 243.0951 | 359.3825 |  |
| [M+H]+ | Simetryn | 1.2242 | 0.3889 | 0.0007 | 214.1121 | 485.4100 |  |
| [M+H]+ | 1-deoxynojirimycin | 2.8339 | 1.6167 | 0.0020 | 164.0707 | 36.7981 |  |
| [M+H]+ | Niacinamide | 9.9042 | 1.6704 | 0.0047 | 123.0556 | 130.8580 |  |
| [M+H]+ | Urocanate | 1.0195 | 2.3264 | 0.0210 | 139.0503 | 338.7515 |  |
| [M+H-H2O]+ | Sulfasalazine | 1.1564 | 1.5126 | 0.0252 | 381.0793 | 405.8640 |  |
| [M+H-C3H6O2]+ | Meperidine | 2.6130 | 0.6439 | 0.0323 | 174.1238 | 509.0440 |  |
| [M+H]+ | 4-hydroxyquinoline | 1.1648 | 1.3399 | 0.0336 | 146.0601 | 245.1735 |  |
| [M+H]+ | Hypoxanthine | 3.4861 | 3.1072 | 0.0337 | 137.0459 | 317.4750 |  |
| [M+H]+ | Uracil | 2.0509 | 1.2860 | 0.0398 | 113.0350 | 288.2875 |  |
| (M+H)+ | Cytosine | 1.2231 | 1.9032 | 0.0448 | 112.0513 | 286.4560 |  |
| [M+H]+ | 1-naphthoic acid | 7.6437 | 3.6698 | 0.0000 | 173.0710 | 106.6015 | Benzenoids |
| [M+H-H2O]+ | Ephedrine | 2.3836 | 0.6155 | 0.0001 | 148.0969 | 354.1055 |  |
| [M+H]+ | 4-amino-2,6-dinitrotoluene | 1.1010 | 0.4183 | 0.0004 | 198.0696 | 424.8430 |  |
| [M+H]+ | Plumbagine | 3.3683 | 3.7143 | 0.0006 | 189.0659 | 74.5102 |  |
| [M+Na]+ | Isoproturon | 1.5725 | 2.1198 | 0.0011 | 229.1183 | 391.0340 |  |
| [M+H-CH6O2]+ | Dl-normetanephrine | 2.0310 | 1.6733 | 0.0014 | 134.0602 | 39.3698 |  |
| [M+H-2H2O]+ | 4-hydroxyphenethyl alcohol | 1.3597 | 0.7371 | 0.0020 | 103.0547 | 326.3390 |  |
| [M+H]+ | (1e,4e)-1,5-bis(4-methoxyphenyl)penta-1,4-dien-3-one | 1.1163 | 0.6089 | 0.0036 | 295.1174 | 63.6441 |  |
| [M+H]+ | Methiocarb sulfoxide | 1.0322 | 1.6609 | 0.0129 | 242.1053 | 899.9155 |  |
| [M+H]+ | Butamifos | 1.0214 | 1.7861 | 0.0177 | 333.0790 | 359.0190 |  |
| [M+H-C8H4F3O2N]+ | Triflumuron | 1.3049 | 1.2417 | 0.0445 | 156.0422 | 344.9150 |  |
| [M+H-C2H5N]+ | Cytisine | 2.2533 | 3.1731 | 0.0001 | 148.0757 | 38.6557 | Alkaloids and derivatives |
| [M]+ | Sanguinarine | 1.3509 | 1.8278 | 0.0025 | 332.0950 | 359.4940 |  |
| [M+H]+ | Oxymatrine | 1.8846 | 0.0085 | 0.0209 | 265.1911 | 190.6755 |  |
| [M+Na]+ | Lactose | 6.0501 | 1.7970 | 0.0028 | 365.1056 | 405.7930 | Organic oxygen compounds |
| [2 M+Na]+ | 4-alpha-mannobiose | 1.2395 | 2.9299 | 0.0038 | 707.2221 | 405.5920 |  |
| [M+H]+ | 2-hexenal | 1.0251 | 0.5803 | 0.0120 | 99.0922 | 532.8355 |  |
| [M+H]+ | 2,4-dimethylmethcathinone | 1.0232 | 0.4865 | 0.0321 | 192.1595 | 109.1600 |  |
| [M+H]+ | Phencyclidine | 1.0403 | 0.5529 | 0.0017 | 244.1907 | 236.6270 | Organic nitrogen compounds |
| [M+H]+ | Diethanolamine | 1.5890 | 0.6387 | 0.0257 | 106.0867 | 398.8210 |  |
| [M-H]- | Guanidinosuccinic acid | 1.3882 | 5.6656 | 0.0000 | 174.0556 | 67.7538 | Organic acids and derivatives |
| [M-H]- | 2-ketohexanoic acid | 2.4474 | 0.0497 | 0.0002 | 129.0549 | 319.3190 |  |
| [M-H]- | Phenaceturic acid | 6.6140 | 1.9861 | 0.0003 | 192.0665 | 281.8215 |  |
| [M-H]- | Acetaminophen sulfate | 1.2325 | 2.4825 | 0.0004 | 230.0148 | 37.7345 |  |
| [M-H]- | N-(phosphonomethyl)glycine | 1.2350 | 0.1293 | 0.0005 | 168.0295 | 101.4590 |  |
| [M-H]- | Ketoleucine | 5.5080 | 0.6378 | 0.0008 | 129.0549 | 97.0668 |  |
| [M-H]- | Maleic acid | 1.5101 | 0.3655 | 0.0014 | 115.0028 | 305.4020 |  |
| (M-H)- | 3-Methyl-2-oxopentanoate | 5.6112 | 0.3899 | 0.0019 | 129.0549 | 152.8800 |  |
| [M-H]- | Glycine | 1.7481 | 1.9284 | 0.0025 | 74.0236 | 281.8570 |  |
| [M-H]- | Bonactin | 1.0213 | 0.1302 | 0.0041 | 399.2387 | 270.5720 |  |
| [M-H]- | Isoleucine | 2.7855 | 0.7238 | 0.0083 | 130.0865 | 338.7820 |  |
| [M-H]- | 3-hydroxybutyric acid | 3.0433 | 0.6532 | 0.0236 | 103.0390 | 324.1110 |  |
| [M-H]- | D-ornithine | 1.1106 | 1.3839 | 0.0359 | 131.0817 | 399.6950 |  |
| [M-H]- | Sarcosine | 1.8136 | 0.8195 | 0.0413 | 88.0393 | 385.2330 |  |
| [M-H]- | 12s-hydroxy-5z,8z,10e,14z-eicosatetraenoic acid | 2.1899 | 10.7129 | 0.0000 | 319.2282 | 104.1645 | Lipids and lipid-like molecules |
| [M-H]- | Lithocholylglycine | 1.3217 | 3.4460 | 0.0000 | 432.3130 | 260.3850 |  |
| [2 M-H]- | 2-cis-4-trans-abscisic acid | 1.3460 | 6.1342 | 0.0000 | 527.2687 | 59.4630 |  |
| [M-H]- | Hexacosanoic acid | 1.1262 | 2.2406 | 0.0001 | 395.3905 | 64.2367 |  |
| [M-H]- | Arachidonic acid (peroxide free) | 3.2981 | 2.2053 | 0.0002 | 303.2332 | 64.2509 |  |
| [M-H]- | 5-alpha-pregnan-3-alpha.,17-diol-20-one 3-sulfate | 1.1311 | 0.0184 | 0.0003 | 413.2193 | 292.8440 |  |
| [M+Cl]- | 7-keto-3-alpha,12-alpha.-dihydroxycholanic acid | 3.5477 | 6.0693 | 0.0004 | 441.2426 | 284.0940 |  |
| [M-H-O3S]- | Taurolithocholic acid sulfate | 1.2108 | 1.9201 | 0.0006 | 482.2950 | 63.5875 |  |
| [M-H]- | Nonanoic acid | 2.7373 | 1.4305 | 0.0010 | 157.1228 | 105.4235 |  |
| [M-H]- | 16-hydroxyhexadecanoic acid | 1.7275 | 0.4424 | 0.0016 | 271.2280 | 64.2589 |  |
| [M-H]- | Linoleic acid | 5.9993 | 0.4732 | 0.0047 | 279.2333 | 64.5071 |  |
| [M-H]- | 11-dehydrothromboxane b2 | 2.1890 | 1.9212 | 0.0086 | 367.2158 | 63.4337 |  |
| [M-H]- | Eicosenoic acid | 1.4829 | 0.2981 | 0.0197 | 309.2801 | 64.5071 |  |
| [M-H]- | Periplogenin | 1.1083 | 0.5691 | 0.0216 | 389.2190 | 305.8390 |  |
| [M-H]- | Heptadecanoic acid | 4.0713 | 0.3370 | 0.0239 | 269.2487 | 64.8139 |  |
| [M-H]- | Linolenic acid | 1.9198 | 0.5442 | 0.0242 | 277.2177 | 64.5071 |  |
| [M-H]- | Oleic acid | 16.5792 | 0.2307 | 0.0280 | 281.2490 | 64.4983 |  |
| [M-H]- | Lpe 18:2 | 1.5808 | 0.6454 | 0.0284 | 476.2792 | 283.4770 |  |
| [M-H]- | Ethyl laurate | 3.2633 | 0.3434 | 0.0309 | 227.2017 | 65.2703 |  |
| [M-H]- | Glycodeoxycholic acid | 5.3723 | 1.7042 | 0.0342 | 448.3080 | 283.4125 |  |
| [M-H]- | (z)-5,8,11-trihydroxyoctadec-9-enoic acid | 1.1062 | 0.5877 | 0.0353 | 329.2478 | 64.2509 | Lipids and lipid-like molecules |
| [M-H-C7H6O8]- | Dehydro-l-(+)-ascorbic acid dimer | 1.6653 | 0.2793 | 0.0005 | 129.0187 | 240.8600 | Organoheterocyclic compounds |
| [M-H]- | 5-azauracil | 1.5988 | 0.5231 | 0.0134 | 112.0032 | 212.5880 |  |
| [M-H]- | Adenine | 1.9410 | 1.3682 | 0.0229 | 134.0602 | 288.2810 |  |
| [M+OH]- | L-gulono-1,4-lactone | 1.2729 | 1.3808 | 0.0395 | 195.0507 | 370.3870 |  |
| [M-H]- | Phenol | 7.8491 | 0.0645 | 0.0000 | 93.0335 | 59.8854 | Benzenoids |
| [M-H]- | Salicyluric acid | 9.2290 | 0.0851 | 0.0000 | 194.0458 | 264.7925 |  |
| [M-H+2i]- | Fenhexamid | 1.8509 | 0.1203 | 0.0001 | 302.0495 | 34.5489 |  |
| [M-H]- | 2,6-dihydroxybenzoic acid | 3.6693 | 0.1531 | 0.0007 | 153.0186 | 37.5110 |  |
| (M-H)- | 4-Hydroxybenzoate | 1.8504 | 0.1540 | 0.0013 | 137.0237 | 209.3610 |  |
| [M-H]- | Zinniol | 1.5889 | 0.6878 | 0.0034 | 265.1489 | 36.3976 |  |
| [M-H]- | 2,3-dihydroxybenzoic acid | 1.6846 | 0.1290 | 0.0039 | 153.0186 | 287.2710 |  |
| [M-H-CO2]- | 3-hydroxyphenylacetic acid | 6.1555 | 1.8903 | 0.0067 | 107.0492 | 35.0010 |  |
| [M-H]- | Hippuric acid | 6.7623 | 1.3897 | 0.0209 | 178.0503 | 288.2810 |  |
| [M-H-2H2O]- | Shikimate | 13.5828 | 0.0679 | 0.0000 | 137.0237 | 59.9250 | Organic oxygen compounds |
| [M-H]- | D-glucosamine, 6-sulfate | 2.5448 | 6.1181 | 0.0000 | 258.0081 | 58.1261 |  |
| [M-H]- | Uridine | 1.1409 | 1.4339 | 0.0191 | 243.0624 | 293.8620 | Nucleosides nucleotides and analogs |
| [M-H]- | Rhamnetin | 2.3272 | 0.0512 | 0.0001 | 315.0549 | 53.9841 | Phenylpropanoids and polyketides |

Adduct represents the adduction information of the compound; Name represents the name of the metabolite; VIP represents the variable projection importance; FC represents the difference fold; p-value represents the P value of the significance analysis; m/z means the mass-to-charge ratio; rt(s) means the retention time of the metabolite on the chromatogram, that is, the peak time, in seconds; Superclass is the classification of the corresponding metabolite;

**Table S3** Raw date of ruminal fermentation parameters

| Group | Cow | Acetate | Propionate | Butyrate | A/P | NH3-N |
| --- | --- | --- | --- | --- | --- | --- |
| Group G | 0706 | 36.391 | 21.397 | 1.843 | 1.700752442 | 3.500 |
| Group G | 0704 | 37.159 | 24.932 | 2.111 | 1.490413926 | 3.167 |
| Group G | 0689 | 36.744 | 27.103 | 3.175 | 1.355717079 | 2.167 |
| Group B | 6175 | 49.024 | 37.124 | 2.641 | 1.320547355 | 6.833 |
| Group B | 5123 | 57.426 | 35.993 | 2.602 | 1.595476898 | 7.833 |
| Group B | 2004 | 64.723 | 36.569 | 2.697 | 1.769887063 | 4.833 |

**Table S4** Raw date of ruminal microbes alpha index

| Group | Cow | Chao1 | Observed_species | Shannon | Simpson |
| --- | --- | --- | --- | --- | --- |
| Group G | 0704 | 5332.8 | 3918.8 | 10.4583 | 0.99803 |
| Group G | 0689 | 5315.61 | 3848.3 | 10.5324 | 0.998303 |
| Group G | 0706 | 5266.55 | 3744.1 | 10.3938 | 0.997879 |
| Group B | 6175 | 4401.44 | 3374.4 | 10.328 | 0.997925 |
| Group B | 5123 | 2894.36 | 2125.7 | 6.29303 | 0.787554 |
| Group B | 2004 | 4248.97 | 3273.8 | 10.3236 | 0.998192 |

**Table S5** Raw date of Serum antioxidant indices

| Group | TAOC | MDA | SOD | GSH-Px |
| --- | --- | --- | --- | --- |
| G1-1 | 5.893 | 1.649 | 71.636 | 205.459 |
| G1-2 | 5.875 | 1.406 | 76.513 | 202.141 |
| G2-1 | 5.736 | 2.606 | 36.864 | 179.718 |
| G2-2 | 5.734 | 2.526 | 35.462 | 178.192 |
| G3-1 | 5.977 | 2.299 | 84.930 | 219.191 |
| G3-2 | 5.970 | 2.346 | 83.660 | 220.319 |
| G4-1 | 5.780 | 1.799 | 108.997 | 145.088 |
| G4-2 | 5.742 | 1.734 | 107.901 | 146.481 |
| G5-1 | 6.014 | 1.837 | 74.368 | 194.645 |
| G5-2 | 6.045 | 2.023 | 78.937 | 196.038 |
| G6-1 | 5.927 | 1.680 | 29.519 | 202.606 |
| G6-2 | 5.880 | 1.714 | 32.651 | 199.289 |
| B1-1 | 5.865 | 1.481 | 52.326 | 185.689 |
| B1-2 | 5.865 | 1.525 | 49.230 | 189.736 |
| B2-1 | 5.859 | 1.853 | 114.389 | 161.806 |
| B2-2 | 5.907 | 1.729 | 121.481 | 162.801 |
| B3-1 | 5.936 | 1.778 | 87.937 | 216.604 |
| B3-2 | 5.991 | 2.005 | 90.920 | 218.461 |
| B4-1 | 5.761 | 1.698 | 56.185 | 194.380 |
| B4-2 | 5.762 | 1.693 | 53.330 | 191.991 |

**Table S6** Raw date of Serum immune indices

| Group | IL-4 | IL-6 | IL-17 | IgA | IgG | IgM | TNF-α |
| --- | --- | --- | --- | --- | --- | --- | --- |
| G1-1 | 79.753 | 19.356 | 69.228 | 179.317 | 2093.431 | 118.813 | 297.483 |
| G1-2 | 79.379 | 19.370 | 66.124 | 181.969 | 2040.652 | 112.561 | 300.385 |
| G2-1 | 76.713 | 16.355 | 59.784 | 169.810 | 1866.905 | 107.239 | 318.664 |
| G2-2 | 75.853 | 17.178 | 62.284 | 168.621 | 1892.593 | 107.106 | 311.918 |
| G3-1 | 79.467 | 20.040 | 63.411 | 166.565 | 1709.971 | 119.052 | 301.618 |
| G3-2 | 79.269 | 19.835 | 62.398 | 168.484 | 1715.109 | 112.720 | 293.494 |
| G4-1 | 72.174 | 18.116 | 57.513 | 167.890 | 1834.210 | 101.333 | 296.250 |
| G4-2 | 77.175 | 17.485 | 58.183 | 167.204 | 1889.791 | 104.180 | 286.820 |
| G5-1 | 71.887 | 18.490 | 67.888 | 185.625 | 2072.413 | 99.737 | 304.084 |
| G5-2 | 77.109 | 18.212 | 67.463 | 178.952 | 2032.712 | 98.938 | 292.188 |
| G6-1 | 74.157 | 17.671 | 59.392 | 192.756 | 1826.737 | 106.202 | 290.447 |
| G6-2 | 77.704 | 17.317 | 59.179 | 185.945 | 1840.749 | 107.559 | 291.753 |
| B1-1 | 82.552 | 16.647 | 55.617 | 167.890 | 1649.253 | 110.299 | 244.604 |
| B1-2 | 80.282 | 17.384 | 55.960 | 169.627 | 1695.959 | 105.803 | 250.479 |
| B2-1 | 73.386 | 15.121 | 66.777 | 201.395 | 1767.887 | 93.325 | 276.302 |
| B2-2 | 73.606 | 16.231 | 67.578 | 196.824 | 1807.587 | 92.633 | 278.986 |
| B3-1 | 71.997 | 19.117 | 67.627 | 186.722 | 2083.155 | 96.225 | 243.951 |
| B3-2 | 74.465 | 18.499 | 67.414 | 186.768 | 2070.544 | 99.338 | 260.417 |
| B4-1 | 72.372 | 16.690 | 72.512 | 185.351 | 2082.221 | 91.383 | 308.799 |
| B4-2 | 75.479 | 16.820 | 70.780 | 186.357 | 2039.718 | 95.054 | 304.519 |
